# Whole-Genome Sequence of *Mycobacterium bovis* BCG-1 (Russia)

**DOI:** 10.1128/genomeA.01320-15

**Published:** 2015-11-12

**Authors:** R. Ludannyy, M. Alvarez Figueroa, D. Levi, M. Markelov, V. Dedkov, N. Aleksandrova, G. Shipulin

**Affiliations:** a Central Research Institute for Epidemiology of Federal Service on Consumers’ Rights Protection and Human Well-Being Surveillance, Moscow, Russia; b Center for Expertise and Control of Immunobiological Preparation (CEC) of Scientific Center for Expertise of Medical Application Products, Ministry of Health, Moscow, Russia

## Abstract

BCG vaccine (*Mycobacterium bovis* BCG-1 [Russia]) is the most important component of tuberculosis prophylaxis in Russia. This study represents the complete genome sequence and genetic characteristics of *M. bovis* BCG-1 (Russia), which has been used to manufacture BCG vaccine in Russia and in some other countries.

## GENOME ANNOUNCEMENT

*Mycobacterium bovis* BCG remains one of the leading worldwide vaccine strains to date (1). Historically, the ancestral form of BCG—the wild strain *M. bovis*—had been attenuated by serial passages *in vitro* from 1908 to 1921 in Lille and later in Paris at the Pasteur Institute (2, 3). Subsequently, the original strain *M. bovis* BCG was brought from the Pasteur Institute to the CEC of the Scientific Center for Expertise of Medical Application Products (ex. State Control Institute, Russia) and later in 1925 was redistricted as *M. bovis* BCG-I. Since the 1940s *M. bovis* BCG-1 has been preserved by the lyophilization method (4, 5). From 1954 the seed lot system has been adapted in Russia (6). Nowadays, BCG vaccine in Russia is produced from seed lot 368 “shch” (2006).

The molecular phylogenetics of *M. bovis* BCG based on presence or absence of specific regions of difference allowed dividing BCG substrains on two main groups: DU1 and DU2 (7). The complete genomes of BCG substrains Tokyo 172 (8) and Moreau RDJ (9) from DU 2 group I have been applied for comparative genomic analysis in our study.

*M. bovis* BCG-I (Russia), no. 700001 seed lot 368 “shch” (2006), is given by the National State Collection of Pathogenic Microorganisms of Scientific Center for Expertise of Medical Application Products of the Ministry of Health, Russia. The total DNA from *M. bovis* BCG-I was extracted by the classical phenol-chloroform method.

The sample was sequenced on both the Roche GS Junior and Illumina MiSeq NGS platforms (10). For sequencing on the Roche GS Junior, two libraries were prepared using GS Junior sequencing kits, and on the Illumina MiSeq, two libraries were prepared using a Nextera DNA sample prep kit (Illumina). An approximately 35- to 70-fold coverage (*Q* > 25) by Illumina MiSeq was received. Using two sequences from each platform gave rise to higher certainty with low-level variant calls and allowed for the correction of homopolymer regions, mobile elements, and tandem repeats, including gaps and invalid sequences. The reads were collected with Geneious software version 7.0.6 using the MIRA and VELVET plug-ins (11, 12). A total of 318,832 contigs were *de novo* assembled and scaffolded to one circular chromosome. Validation of sequence data was provided by ALE (Assembly Likelihood Evaluation) (13). The automated annotation was performed by Blast2go (14) and verified by Artemis (15). Reannotation was completed by NCBI software. The calculation of genetic distance, selection of reference sequencing, and statistical analysis of genetic data were done with MEGA software (16).

The single circular chromosome of *M. bovis* BCG-I (Russia) is 4,363,945 bp in length, with an average G+C content of 65.5%. The genome of BCG Tokyo (NC_012207.1) was used as a reference sequence for comparative analysis (D = 96.6% [17]). The 4,119 genes were accurately predicted and included 3 rRNAs (16s, 5s, and 23s units organized as typical rDNA cistrons), 45 tRNAs, 34 mobile elements, and 159 tandem and palindromic repeats.

### Nucleotide sequence accession number.

The complete circular genome sequence of vaccine substrain *M. bovis* BCG-1 has been deposited in GenBank under the accession number CP011455.
